# Symptomatic SARS-CoV-2 Infection with Ageusia after Two mRNA Vaccine Doses

**DOI:** 10.3390/ijerph19020886

**Published:** 2022-01-13

**Authors:** Vito Carlo Alberto Caponio, Maria Rosaria Lipsi, Francesca Fortunato, Fabio Arena, Lorenzo Lo Muzio

**Affiliations:** 1 Department of Clinical and Experimental Medicine, University of Foggia, 71122 Foggia, Italy; vitocarlo.caponio@unifg.it (V.C.A.C.); fabio.arena@unifg.it (F.A.); 2 Microbiology and Virology Unit, Policlinico Riuniti Foggia Hospital, 71122 Foggia, Italy; mariarosarialipsi@icloud.com; 3 Hygiene Unit, Policlinico Riuniti Foggia Hospital, 71122 Foggia, Italy; francesca.fortunato@unifg.it; 4 Department of Medical and Surgical Sciences, University of Foggia, 71122 Foggia, Italy; 5 Consorzio Interuniversitario Nazionale per la Bio-Oncologia, 66100 Chieti, Italy

**Keywords:** coronavirus, vaccine, COVID-19, SARS-CoV-2, healthcare worker

## Abstract

To raise awareness about preventive measures in COVID-19 pandemic, even though fully vaccinated. Although recent trials showed high efficacy of vaccines in preventing symptomatic infections, there are some individuals experiencing symptomatic SARS-CoV-2 infection. In this case report, a fully vaccinated young dental practitioner experienced symptomatic SARS-CoV-2 infection 55 days postvaccination with BNT162b2 Pfizer vaccine with evident ageusia. Diagnostic swabs were performed and used for viral genome sequencing. The patient fully recovered 15 days after diagnosis. Loss of smell and taste, together with nasal congestion were the main reported symptoms. The use of personal protective equipment prevented spread of infection in patients and co-workers. With the increase of people being fully vaccinated, it is still necessary to follow infection preventive protocols by correctly applying personal protective equipment. Although high efficacy has been proved, some individuals may still be vulnerable to symptomatic infection and new guidelines and markers should be adopted and investigated to find out patients for whom vaccination may not determine full immunization.

## 1. Introduction

After the SARS-CoV-2 pandemic, the hope of the whole world is placed in the effectiveness of vaccines that should ensure immunity to the entire population. For this reason, the FDA authorized early the new mRNA-based vaccines. Their short-term efficacy has been comparable with that of other common vaccines in phase 3 trials and a reduced symptomatic SARS-CoV-2 infection by over 90% was observed in healthy participants, after vaccination [1,2]. mRNA vaccines were developed to mitigate severe COVID-19 disease [3], showing successful results.

Sometimes the immune system of some subjects does not respond effectively to the vaccination, especially in some categories of patients, such as the immunocompromised. One study, based on solid organ transplant (SOT) recipients, showed reduced humoral response to mRNA vaccines [4].

However, COVID-19 disease after full-dose mRNA vaccination can involve healthy subjects too. A recent study reported eight symptomatic SARS-CoV-2 infections occurring in fully vaccinated health care workers (incidence rate 4.7 per 100,000 person-days adjusted) [5].

We reported, to our knowledge, the first case of an Italian fully vaccinated dental practitioner in whom symptomatic infection emerged. This unique case was described by the patient himself. Transmission of SARS-CoV-2 virus has been implicated in other published reports in fully vaccinated individuals and by publishing several case reports would be useful for understanding COVID-19 disease in vaccinated people, improving prognostication, patients’ selection and general public health policies.

## 2. Case Report

**Figure 1:**
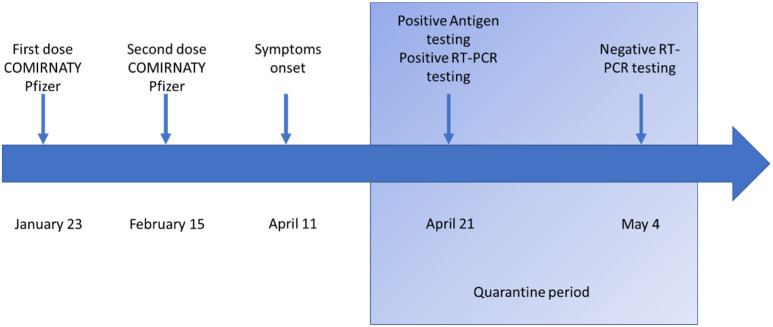
Timeline of events during vaccinations and infection.

A 28-year-old male dentist received the first inoculation of BNT162b2 Pfizer vaccine on 23 January 2021, and the second dose on 15 February (Figure 1). The patient fulfilled general medical requirements for undergoing vaccination and had an overall, generally good health status. No concurrent medical or surgical conditions were reported.

As reported by the patient, on the first dose no adverse events occurred and it was reported only a pain in the arm, where the vaccine was inoculated. Similar symptoms were reported after getting the second dose, without signs of fever, fatigue or more serious events. He spent the second week of 5–11 April 2021, at home, where he met his parents and relatives who had already recovered from COVID-19 from November 2020. Then he moved back to his workplace, at the Dental Clinic of Foggia University. On 11 April, he noted changes in his voice, nasal congestion and symptoms similar to seasonal allergy. On 20 April, he received a call from his aunt stating her positivity to SARS-CoV-2. On 21 April, he was referred to the Infectious Disease and Control Department of the University where he underwent to SARS-CoV-2 antigen test first, and later to RT-PCR test on nasopharyngeal swab. Both tests were positive for new coronavirus infection.

AFIAS COVID-19 Ag (Boditech Med Inc., Chuncheon-si, Korea) was used for the first screening. AFIAS COVID-19 Ag is an automatic fluorescence Immunoassay (FIA) for the qualitative/semiquantitative (through signal intensity cut-off index (COI)) detection of novel coronavirus in human nasopharyngeal swab specimens [6].

Subsequently, viral RNA was extracted within 2 h from the sample collection using the STARMag 96×4 Universal Cartridge kit with the Microlab NIMBUS IVD instrument according to the manufacturer’s instructions (Seegene Inc., Seoul, Korea). Amplification and detection of four target genes N, E, S and RNA-dependent RNA-polymerase was performed using the commercially available kit Allplex SARS-CoV-2 Assay (Seegene Inc., Seoul, Korea) with the CFX96TM instrument (Bio-Rad, Hercules, CA, USA). Results interpretation was performed with the Seegene Viewer software (Seegene, Seoul, Korea).

Sample was therefore used for viral genome sequencing. In brief, total RNA extraction was performed starting from 400 µL of the swab transport medium using a Viral Nucleic Acid Extraction Kit (Low inhibition cartridge 202) with the MagCore workstation. Target enrichment and library preparation for next-generation sequencing was performed using the CleanPlex SARS-CoV-2 Research and Surveillance Panel (Paragon Genomics, Inc., Hayward, CA, USA). Sequencing was performed on a MiSeq Illumina System with a MiSeq v2 reagent kit (300 cycles). Downstream analysis was performed using the SOPHiA DDM software pipeline (Sophia Genetics, Saint-Sulpice, Switzerland). Nextclade software (https://clades.nextstrain.org/, accessed on 21 April 2021) was used to assign clade and call mutations. The obtained genome sequence was deposited at the GISAID global repository (https://www.gisaid.org/, accessed on 22 April 2021) with the EPI_ISL_1890572 Accession ID. According to the currently accepted classification criteria, the viral genome belonged to the VOC Alpha 202012/01 GRY (B.1.1.7) and was characterized by the presence of several known relevant alterations in the S protein (69/70 deletion, 144 deletion, N501Y, A570D, D614G, P681H, T716I, S982A, D1118H).

Patient was quarantined, preventive measures were taken. Specifically, medical and working staff at the dental clinic was also quarantined for one week before undergoing to swab analysis. On 22 April, he reported peak of fever (38 °C) lasting around one hour together with changes in voice and nasal congestion, loss of taste and smell. Paracetamol was administered to control the fever.

On 17 April, patient had already reported to have lost salt taste and nasal congestion, days before testing positive on 21 April. In the following days, nasal congestion decreased, together with recovery of taste and smell. Finally, he was tested negative 4 May, after 14 days of quarantine as recommended by Italian ministerial guidelines. He reported feeling fully recovered even a few days before the last test. Medical and working staff at the dental clinic tested negative even if there was a close contact with the positive patient.

Unfortunately, we did not have data about immune response after vaccination.

## 3. Discussion

Recently, in this scenario, healthy vaccinated subjects report positive tests and symptoms of SARS-CoV-2 infection [7,8], despite confirmation of adequate immune response among these patients [9].

Although more severe susceptibility emerged in patients with obesity, metabolic syndrome, or type 2 diabetes [10,11], alterations in the immune response generated by the vaccination and/or a subsequent contact of a new virus variant, may be the principal cause of such kinds of infection.

Recent studies showed that immunization can be less potent against the beta variant (B.1.351) for a limited protection of neutralizing antibodies created by natural contact with the virus or vaccination [12,13].

In a group of 417 volunteers inoculated with the second dose of BNT162b2 (Pfizer-BioNTech) or mRNA-1273 (Moderna) vaccine, Hacisuleyman et al. identified two women with infection [8]. Genome sequencing highlighted changes with clinical consequences, such as E484K in one female subject and three mutations (T95I, del142–144, and D614G) in both [8]. Both had reported consistent clinical evidence to the vaccine boost, considering the clinical record book, showing neutralizing antibodies, proof of an active immune response to the vaccines.

Indeed, both the patients presented with clinically mild symptoms [8]. Moreover, in patient 1 was observed a high viral load, despite high values of neutralizing antibodies [8]. In this scenario, a risk of illness after fortunate vaccination should be considered because of variant virus infection [14,15,16]. Recently, a study was conducted in New York, including 126,367 fully vaccinated individuals. There were 101 cases of vaccine breakthrough infection between 1 February and 30 April 2021, accounting for 1.4% of the 7147 total SARS-CoV-2 positive cases in their healthcare system and 0.08% of the fully vaccinated population in their medical records; 57 of 76 infections referred to the B.1.1.7 (Alpha) or B.1.526 (Iota) [14].

A recent case report showed the presence of COVID-19 symptoms 40 days postvaccination [7]. Based on the time of the appearance of symptoms and antibody titer, the most plausible cause of the disease was patient’s limited amount of humoral immune response to the vaccine, although no history of immunodeficiency was recorded [7]. Additionally, the antibody level in this patient was much lower than those who previously had COVID-19 [7]. In our case report, the COVID-19 symptoms developed 55 days postvaccination. A previously reported case report supposed that the infection started before the booster shot took full effect [8].

Nowadays, we are still living in an uncertain scenario, in which it is necessary to assess the role of variants in determining postvaccination infection and how the host immune-response may impact in the development of the disease. So, in order to deeply understand these mechanisms, it could be useful, whenever it is possible, to perform viral DNA sequencing and testing for immune-related markers in patients with breakthrough infections.

## 4. Conclusions

This case outlines some useful considerations; firstly, the classic variant of SARS-CoV-2 infected a healthy subject, 55 days postvaccination, pointing out that breakthrough infection is possible after vaccination in healthy subjects with common variants; in addition, moreover, it is necessary to continue observing all the prescriptions recently indicated in the literature in order to avoid new contagion for all healthy workers also after vaccination, since, as seen, the vaccination does not ensure complete immunity in 100% of cases. In this case report, the correct use of personal protective equipment by the dental staff prevented the spread of infection between patients and workers. Personal protective equipment included the use of filtering facepiece 2 (FFP2) and working spaces were arranged to keep a distance of at least 2 m between workers in the office. Each room was also provided with portable air cleaners. In the last instance, we are lacking standardized means to predict a guaranteed immunity status after vaccination. Future research should focus on the development of affordable means for monitoring a population’s immunity postvaccination response in order to adopt preventive measures and understand new mechanisms beyond this particular disease.
